# In vitro antifungal activity and preliminary physicochemical stability of a shampoo containing a hexane extract from *Agave sisalana* residues

**DOI:** 10.1007/s42770-026-02114-4

**Published:** 2026-11-05

**Authors:** Luisa Taynara Silvério da Costa, Julia Amanda Rodrigues Fracasso, Fernando Augusto Araújo Donangelo Oliveira, Amanda Letícia Santos Costa, Wellington Ricardo Pereira Martins, Luisa Cappi de Lima, Beatriz Castro de Silva, Pedro Oliva de Neto, Felipe Romici Zane Lordelo Nogueira, Lucinéia dos Santos

**Affiliations:** 1 Department of Biotechnology, School of Sciences and Languages, São Paulo State University (UNESP), Assis, 19806-900 Brazil; 2 Bioenergy Research Institute (IPBEN), São Paulo State University (UNESP), Assis, 19806- 900 Brazil

**Keywords:** *Agave sisalana*, Agro-industrial residue, Antifungal activity, *Malassezia pachydermatis*, *Trichophyton rubrum*, Topical formulation

## Abstract

*Agave sisalana* processing generates large amounts of leaf residue that may represent a source of biologically active compounds. This study evaluated the in vitro antifungal activity of a hexane extract obtained from *A. sisalana* residue (HES) and the preliminary physicochemical stability of an HES-containing shampoo. The minimum inhibitory concentrations (MICs) of HES and ketoconazole were determined against *Malassezia pachydermatis* ATCC 14,522 and *Trichophyton rubrum* ATCC 28,189. The antifungal activity of the HES-containing shampoo against *T. rubrum* was subsequently evaluated by viable-count assays after 30, 60, and 90 min of contact. The formulation was also monitored for appearance, odor, homogeneity, pH, and viscosity during a 30-day stability study. HES showed MICs of 125 µg mL⁻¹ against *M. pachydermatis* and 750 µg mL⁻¹ against *T. rubrum*, whereas the corresponding MICs of ketoconazole were 112.5 and 900 µg mL⁻¹, respectively. Across the evaluated contact times, the HES-containing shampoo produced significantly lower colony-forming unit counts than the shampoo base (*p* < 0.001). No statistically significant difference was detected between the HES-containing and ketoconazole-containing shampoos (*p* = 0.564); however, this finding was not interpreted as evidence of statistical equivalence. No changes in appearance, odor, or homogeneity were observed throughout the 30-day evaluation. The pH remained approximately 6.0 under the evaluated storage conditions, while viscosity remained approximately 3000 cP at 25 °C. These findings support the in vitro antifungal activity of HES and warrant further investigation of the formulation, including chemical characterization of the extract, safety assessment, and longer-term stability studies.

## Introduction

Brazil is a major producer of hard fibers obtained from *Agave sisalana* (sisal), which are used in the manufacture of ropes, twine, carpets, sacks, upholstery, automotive components, and handicrafts [1–4]. However, fibers account for only approximately 5% of the fresh leaf mass, whereas the remaining material constitutes an agro-industrial residue that is frequently discarded in the production areas [5, 6]. The recovery of biologically active compounds from this residue may therefore contribute to its valorization and support the development of circular bioeconomy strategies.

Phytochemical investigations of *A. sisalana* have reported the presence of steroidal saponins and sapogenins in different plant-derived fractions [5, 7, 8]. Compounds belonging to these chemical classes have been associated with several biological activities, including antifungal effects [9, 10]. Nevertheless, the composition and biological activity of plant extracts depend on the raw material and extraction conditions. Therefore, the antifungal activity of a specific fraction cannot be attributed to saponins or any other chemical class without appropriate chemical characterization.

Dermatophytoses are superficial fungal infections involving keratinized tissues, including the skin, hair, and nails [11, 12]. *Trichophyton rubrum* is one of the most prevalent anthropophilic dermatophytes and an important etiological agent of human dermatophytosis in Brazil and worldwide [13, 14]. In contrast, *Malassezia pachydermatis* is a commensal yeast of the skin and mucosal surfaces of animals, particularly dogs, but its excessive proliferation under favorable conditions may be associated with dermatitis and otitis [15]. These microorganisms differ in morphology, physiology, nutritional requirements, and susceptibility to antifungal agents, making them relevant models for the preliminary assessment of antifungal activity.

The management of superficial fungal infections may require prolonged topical or systemic therapy. Recurrence, treatment failure, and the emergence of antifungal resistance have increased interest in the identification of compounds with activity against dermatophytes and opportunistic yeasts [13, 16–18]. Plant-derived extracts and isolated natural compounds have consequently been investigated as sources of antifungal agents with different chemical structures and potential mechanisms of action. However, antifungal activity demonstrated in vitro does not establish clinical efficacy or safety and should be regarded as an initial step in the evaluation of a candidate substance.

A previous study demonstrated the antifungal activity of an aqueous extract obtained from *A. sisalana* residue against *Candida albicans* and *M. pachydermatis* [5]. Nevertheless, the antifungal activity of less polar fractions obtained from this residue and their incorporation into topical formulations remain insufficiently investigated. Hexane partitioning provides a fraction enriched in compounds with greater affinity for the organic phase, although chemical characterization is required to determine its actual composition. Furthermore, the incorporation of a plant-derived extract into a shampoo requires evaluation of whether its antifungal activity is retained after incorporation into the formulation and whether the formulation maintains its physicochemical characteristics during storage [19–21].

Therefore, this study aimed to obtain a hexane extract from *A. sisalana* residue (HES) and determine its in vitro antifungal activity against *M. pachydermatis* and *T. rubrum*. An HES-containing shampoo was subsequently formulated, and its in vitro antifungal activity against *T. rubrum*, as well as its preliminary organoleptic and physicochemical stability over 30 days, was evaluated.

## Methods

### Plant material collection

The sisal residue generated during the mechanical removal of hard fibers from the leaves (defibering) was obtained from rural producers in the municipality of Valente, Bahia, Brazil (latitude − 11.41334759; longitude − 39.4653165). The plant species was identified as *Agave sisalana* Perrine at the Herbarium Assisense of São Paulo State University (UNESP), Assis, São Paulo, Brazil, where a voucher specimen was deposited under accession number 2597.

### Obtaining the hexane extract (HES)

**Fig. 1:**
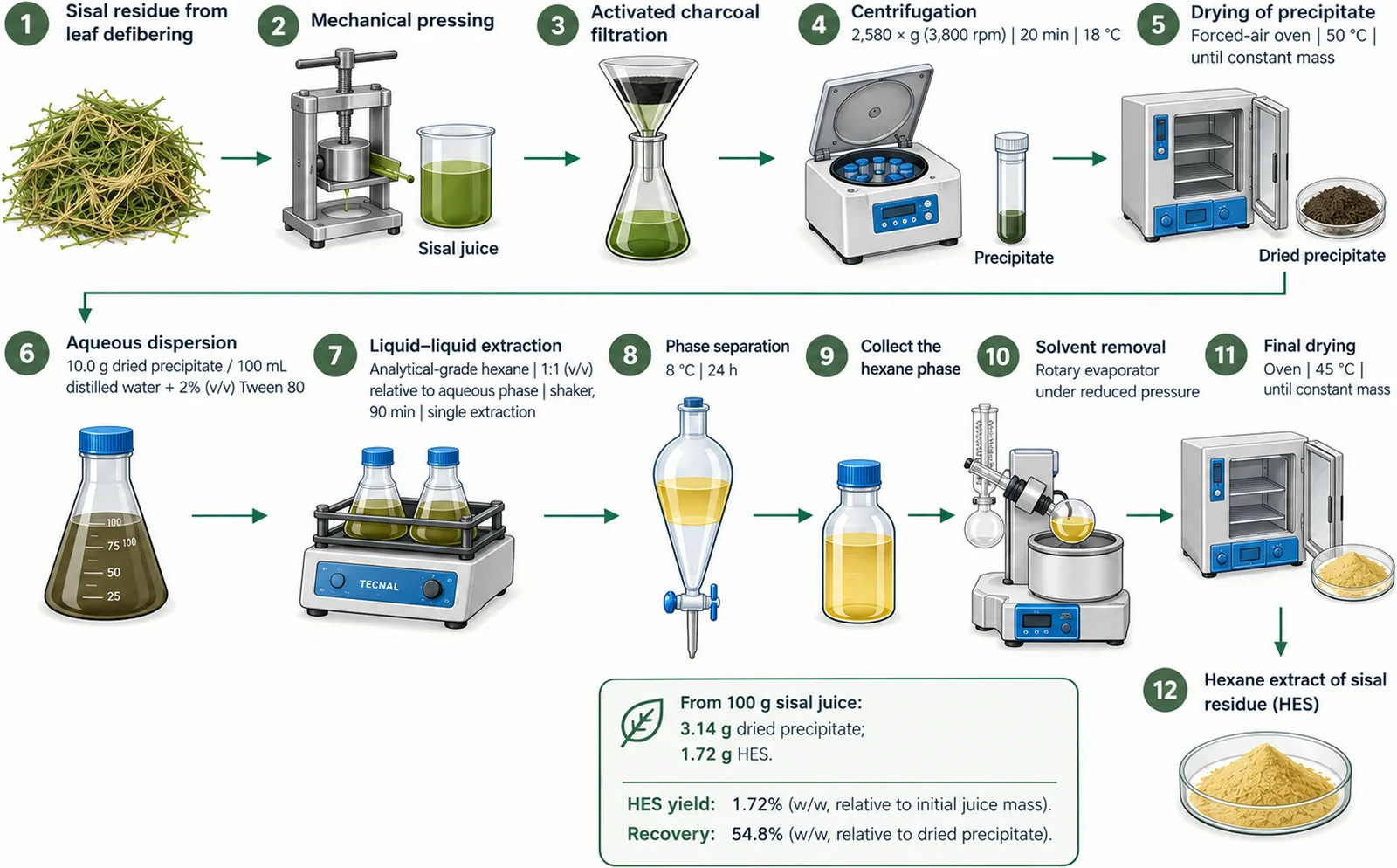
Flowchart of the procedure used to obtain the hexane extract from *Agave sisalana* residue (HES)

Sisal juice was obtained by mechanically pressing the residue generated during leaf defibering. The juice was filtered using activated charcoal and centrifuged (Megafuge 16R, Thermo Scientific) at approximately 2,580 ×g (3,800 rpm) for 20 min at 18 °C to recover the precipitated material. The precipitate was dried in a forced-air oven (Fanem Ltda., model 002CB) at 50 °C until a constant mass was obtained (Fig. 1).

The dried precipitate was dispersed at a proportion of 10.0 g per 100 mL of distilled water containing Tween 80 at 2% (v/v). Tween 80 was used as a dispersing agent to promote homogeneous dispersion of the dried material in the aqueous phase before liquid–liquid partitioning.

Analytical-grade hexane was added to the aqueous dispersion at a 1:1 volume ratio relative to the distilled water. The mixture was agitated in a shaker (Tecnal, model TE-420) for 90 min in a single extraction step and subsequently transferred to a separatory funnel. The system was maintained at 8 °C for 24 h to allow phase separation. The hexane phase was collected and concentrated under reduced pressure using a rotary evaporator for solvent removal. The resulting material was dried in an oven at 45 °C until a constant mass was obtained, yielding the hexane extract of sisal residue (HES).

Under these conditions, 100 g of sisal juice yielded 3.14 g of dried precipitate and subsequently 1.72 g of HES. The HES extraction yield, calculated relative to the initial mass of sisal juice, was 1.72% (w/w). The recovery of HES relative to the dried precipitate was 54.8% (w/w).

$$\begin{array}{c}\mathrm{Extraction}\;\mathrm{yield}\;(\%)\\=\left(\mathrm{mass}\;\mathrm{of}\;\mathrm{HES}/\mathrm{mass}\;\mathrm{of}\;\mathrm{sisal}\;\mathrm{juice}\right)\times100\end{array}$$

$$\begin{array}{c}\mathrm{Recovery}\;\mathrm{from}\;\mathrm{the}\;\mathrm{dried}\;\mathrm{precipitate}\;(\%)\\=(\mathrm{mass}\;\mathrm{of}\;\mathrm{HES}/\mathrm{mass}\;\mathrm{of}\;\mathrm{dried}\;\mathrm{precipitate})\times100\end{array}$$

### Evaluation of the in vitro antifungal activity of HES

#### Fungal strains

The in vitro antifungal activity of HES was evaluated against the reference strains *Malassezia pachydermatis* ATCC 14,522 and *Trichophyton rubrum* ATCC 28,189. Both strains were obtained from the Tropical Culture Collection – André Tosello Foundation (Campinas, São Paulo, Brazil).

#### Maintenance and activation of the fungal strains

The fungal strains were cultivated in modified malt medium containing malt extract (1.8%, w/v), peptone (1.8%, w/v), Tween 80 (0.5%, v/v), glycerol (0.25%, v/v), and chloramphenicol (0.009%, w/v). The medium was prepared with distilled water, adjusted to pH 6.5, and sterilized at 121 °C for 20 min. After inoculation, the cultures were incubated at 32 °C for 72 h.

For preservation, aliquots of each culture were transferred to cryovials containing sterile glycerol at a final concentration of 10% (v/v). The cryovials were stored in liquid nitrogen and at − 20 °C until use.

For activation, cryopreserved cultures were aseptically transferred to Erlenmeyer flasks containing 100 mL of liquid medium composed of sucrose (2.0%, w/v), yeast extract (0.3%, w/v), malt extract (0.3%, w/v), and peptone (0.5%, w/v), adjusted to pH 6.5. The cultures were incubated at 32 °C under agitation at 180 rpm for 5 days. Two sequential subcultures were subsequently performed under the same conditions before preparation of the fungal inocula.

#### Preparation and standardization of the fungal inocula

For preparation of the *M. pachydermatis* inoculum, the activated culture was inoculated onto Sabouraud dextrose agar supplemented with olive oil (1%, v/v) and incubated at 37 °C for 7 days. Yeast cells were harvested from the agar surface and suspended in sterile distilled water. The cell concentration was determined using a Neubauer counting chamber and adjusted to 1 × 10⁶ cells mL⁻¹.

For preparation of the *T. rubrum* inoculum, the activated culture was grown on Sabouraud dextrose agar at 35 °C for 5 days. Sterile saline solution (0.9% NaCl) containing Tween 20 (0.05%, v/v) was added to the agar surface, which was gently scraped to release the conidia. The resulting suspension was homogenized using a tube mixer for 30 s and allowed to stand undisturbed for 10 min to promote the sedimentation of larger hyphal fragments and mycelial aggregates. The upper portion of the suspension, containing predominantly free conidia, was carefully collected without disturbing the sediment. The conidial concentration was determined using a Neubauer counting chamber and adjusted to 1 × 10⁶ conidia mL⁻¹.

The standardized inoculum suspensions were used immediately in the antifungal susceptibility assays.

#### Minimum inhibitory concentration (MIC)

The antifungal activity of HES was evaluated directly using a broth macrodilution assay based on the method described by Artis et al. [22], with modifications. A formal agar-diffusion screening assay was not included because the lipophilic nature of HES could result in limited and non-uniform diffusion through the solid medium. Broth macrodilution was therefore selected to promote more homogeneous contact between HES and the fungal inoculum and to allow the evaluation of a defined concentration range.

The MIC was defined as the lowest final concentration of HES or ketoconazole that completely prevented visible fungal growth under the experimental conditions.

HES and ketoconazole solutions were prepared as twofold serial dilutions at twice the intended final assay concentrations. For *M. pachydermatis*, HES solutions were prepared at 1,000, 500, 250, 125, and 62.5 µg mL⁻¹. For *T. rubrum*, HES solutions were prepared at 3,000, 1,500, 750, 375, and 187.5 µg mL⁻¹. Ketoconazole solutions were prepared at 3,600, 1,800, 900, 450, 225, and 112.5 µg mL⁻¹ for both microorganisms.

For each concentration, 4 mL of the HES or ketoconazole solution was mixed with 4 mL of the appropriate liquid culture medium containing the standardized fungal inoculum at 1 × 10⁶ cells mL⁻¹ for *M. pachydermatis* or 1 × 10⁶ conidia mL⁻¹ for *T. rubrum*. Consequently, the final inoculum concentration in each assay tube was 5 × 10⁵ cells or conidia mL⁻¹.

After the 1:1 dilution with the inoculated culture medium, the final HES concentrations were 500, 250, 125, 62.5, and 31.25 µg mL⁻¹ for *M. pachydermatis* and 1,500, 750, 375, 187.5, and 93.75 µg mL⁻¹ for *T. rubrum*. The final ketoconazole concentrations were 1,800, 900, 450, 225, 112.5, and 56.25 µg mL⁻¹ for both microorganisms.

Growth-control tubes contained the standardized fungal inoculum and culture medium without HES or ketoconazole. Vehicle-control tubes contained the standardized inoculum, culture medium, and Tween 80 at the same final concentration present in the HES treatments. Sterility controls containing culture medium without fungal inoculum were also included.

The tubes inoculated with *M. pachydermatis* were incubated at 37 °C for 72 h. The tubes inoculated with *T. rubrum* were incubated at 35 °C for 5 days. Fungal growth was assessed visually by comparison with the growth and sterility controls. The MIC corresponded to the lowest final concentration showing complete absence of visible fungal growth. The assays were performed in three independent experiments.

### Preparation of the shampoo formulations

The concentration of HES incorporated into the shampoo was selected using the ratio between the concentration of ketoconazole in a commercial 2% (w/v) shampoo and the MIC of ketoconazole against *T. rubrum* determined under the experimental conditions of this study. The ketoconazole concentration in the commercial shampoo, 20 mg mL⁻¹, was approximately 22 times higher than the ketoconazole MIC of 900 µg mL⁻¹. The same rounded factor was applied to the HES MIC of 750 µg mL⁻¹, resulting in a selected HES concentration of 16.5 mg mL⁻¹. Therefore, 1.65 g of HES was incorporated into 100 mL of the shampoo formulation. This proportionality was used exclusively as an experimental criterion for selecting the HES concentration and was not interpreted as evidence of therapeutic or statistical equivalence between HES and ketoconazole.

The composition of the HES-containing shampoo is presented in Table 1. The shampoo base had the same composition but did not contain HES.

**Table 1:** Composition of the HES-containing shampoo formulation

Component	Amount
Sodium lauryl sulfate	23.0 g
Coconut fatty acid diethanolamide	4.0 g
Methylparaben	0.2 g
Disodium EDTA	0.1 g
HES	1.65 g
Sodium chloride solution (20%, w/v)	q.s.
Citric acid solution (1%, w/v)	q.s.
Purified water	q.s. to 100 mL

The shampoo base was prepared at room temperature by combining the components under continuous stirring until a visually homogeneous formulation was obtained. For preparation of the HES-containing shampoo, HES was added directly to the shampoo base and mixed at room temperature until visually homogeneous. Because the stirring time and speed were not recorded, these parameters are not reported.

A 20% (w/v) sodium chloride solution was gradually added to adjust the viscosity to approximately 3,000 cP. The pH was subsequently adjusted to 6.0–6.5 by the gradual addition of a 1% (w/v) citric acid solution. The final volume was adjusted to 100 mL with purified water, and the formulation was homogenized.

Three independent batches of the shampoo base and three independent batches of the HES-containing shampoo were prepared. The shampoo base without HES was used as the formulation control. A commercially available shampoo containing ketoconazole at 20 mg mL⁻¹ was used directly as the reference formulation in the subsequent in vitro antifungal assay.

### In vitro antifungal activity of the shampoo formulations

For the evaluation of the in vitro antifungal activity of the shampoo formulations, *T. rubrum* was cultivated in liquid medium at 30 °C for up to 48 h. A concentrated conidial suspension was obtained and quantified using a Neubauer counting chamber. The concentration was adjusted to approximately 3.7 × 10⁸ conidia mL⁻¹.

An aliquot of 1 mL of the conidial suspension was added to 9 mL of each tested formulation: shampoo base, HES-containing shampoo, and commercial ketoconazole-containing shampoo. This 1:10 dilution resulted in an initial fungal concentration of approximately 3.7 × 10⁷ conidia mL⁻¹ in each treatment. The mixtures were homogenized and maintained at room temperature during the contact period.

After contact times of 30, 60, and 90 min, aliquots from each treatment were immediately subjected to decimal serial dilution in sterile 0.1% (w/v) peptone water. Dilutions were performed until an estimated concentration of 3.7 × 10² conidia mL⁻¹ was obtained. An aliquot of 100 µL from the selected dilution was plated onto Sabouraud dextrose agar supplemented with chloramphenicol at 0.018% (w/v).

The plates were incubated at 35 °C. The first colony count was performed after 24 h of incubation, followed by successive counts until no additional colonies were observed. Colony-forming units were expressed as CFU mL⁻¹ and calculated by considering the number of colonies, the reciprocal of the dilution factor, and the plated volume.

Three independent experiments were conducted for each treatment and contact time. Each independent experiment used one of the three independently prepared batches of the shampoo base and HES-containing shampoo. The commercial ketoconazole-containing shampoo was used as received as the reference formulation.

### Preliminary physicochemical stability evaluation

The preliminary physicochemical stability of the HES-containing shampoo was evaluated over 30 days using three independently prepared batches. Samples from each batch were stored at 4 °C, 25 °C, and 40 °C. The evaluation included organoleptic characteristics, pH, and viscosity. This short-term study was designed as a preliminary stability assessment and was not intended to establish the shelf life of the formulation.

#### Organoleptic characteristics

The appearance, color, odor, homogeneity, and occurrence of visible phase separation were evaluated at the beginning of the study and weekly throughout the 30-day storage period. Appearance, color, homogeneity, and phase separation were assessed visually, whereas odor was evaluated by direct olfactory examination. The samples maintained at 4 °C, 25 °C, and 40 °C were compared with their respective initial characteristics.

#### pH

For pH determination, the shampoo samples were diluted 1:10 (v/v) in distilled water and homogenized before measurement. The pH was measured using a digital pH meter (Tecnal, São Paulo, Brazil). Measurements were performed in triplicate for each independent batch at the beginning of the study, daily during the first 4 days, and weekly thereafter until day 30. Samples stored at 4 °C, 25 °C, and 40 °C were evaluated.

#### Viscosity

Viscosity was determined using a rotational viscometer (Brookfield Programmable DV-II+ Viscometer) and expressed in centipoise (cP). Measurements were performed for the samples maintained at 25 °C at the beginning of the study, daily during the first 4 days, and weekly thereafter until day 30. The three independently prepared batches were evaluated.

### Statistical analysis

Colony-forming unit counts obtained in the shampoo antifungal activity assay were transformed as log₁₀(CFU mL⁻¹ + 1) before statistical analysis. The three independent experiments, corresponding to the three independently prepared formulation batches, were considered the experimental replicates.

The transformed data were analysed using a two-way analysis of variance (ANOVA), with shampoo treatment and contact time as fixed factors, including the treatment × contact time interaction. Homogeneity of variances was assessed using Levene’s test. When a significant treatment effect was detected, pairwise comparisons were performed using Tukey’s honestly significant difference test. The significance level was set at 5% (α = 0.05), and partial eta squared (η²p) was calculated as a measure of effect size.

The MIC results were analysed descriptively because the endpoint was based on the qualitative absence or presence of visible fungal growth. Data from the preliminary stability evaluation were summarized using the mean and standard deviation of the three independent batches.

All statistical analyses were performed using Python version 3.12 and the Pingouin statistical package version 0.5.

## Results

### Antifungal activity of HES

HES completely inhibited the visible growth of *M. pachydermatis* at final concentrations ranging from 125 to 500 µg mL⁻¹, resulting in an MIC of 125 µg mL⁻¹. Ketoconazole completely inhibited visible growth at concentrations ranging from 112.5 to 1,800 µg mL⁻¹, with an MIC of 112.5 µg mL⁻¹ (Table 2).

**Table 2:** Determination of the minimum inhibitory concentration (MIC) of ketoconazole and HES against *Malassezia pachydermatis*

Treatment	Final concentration (µg mL⁻¹)	Visible fungal growth
Ketoconazole	1,800	No
	900	No
	450	No
	225	No
	112.5	No*
	56.25	Yes
HES	500	No
	250	No
	125	No*
	62.5	Yes
	31.25	Yes

*No* complete absence of visible fungal growth, *Yes* visible fungal growth. *MIC defined as the lowest final concentration that completely prevented visible fungal growth. Results were consistent across three independent experiments

For *T. rubrum*, complete inhibition of visible growth was observed at HES concentrations of 750 and 1,500 µg mL⁻¹, resulting in an MIC of 750 µg mL⁻¹. Ketoconazole completely inhibited visible growth at 900 and 1,800 µg mL⁻¹, with an MIC of 900 µg mL⁻¹ (Table 3). Thus, under the experimental conditions used, higher concentrations of both HES and ketoconazole were required to prevent the visible growth of *T. rubrum* than of *M. pachydermatis*. The results were consistent across three independent experiments.

**Table 3:** Determination of the minimum inhibitory concentration (MIC) of ketoconazole and HES against *Trichophyton rubrum*

Treatment	Final concentration (µg mL⁻¹)	Visible fungal growth
Ketoconazole	1,800	No
	900	No*
	450	Yes
	225	Yes
	112.5	Yes
	56.25	Yes
HES	1,500	No
	750	No*
	375	Yes
	187.5	Yes
	93.75	Yes

*No* complete absence of visible fungal growth, *Yes* visible fungal growth. *MIC defined as the lowest final concentration that completely prevented visible fungal growth. Results were consistent across three independent experiments

### Evaluation of the antifungal activity of the HES-containing shampoo

The mean *T. rubrum* CFU counts obtained after contact with the shampoo formulations are presented in Table 4. Two-way ANOVA revealed a significant effect of treatment (*F*(2, 18) = 21.38, *p* < 0.001, partial η² = 0.70). No significant effect of contact time (*p* = 0.474) or treatment × contact time interaction (*p* = 0.694) was detected.

**Table 4:** Mean *Trichophyton rubrum* counts after contact with the shampoo base, ketoconazole-containing shampoo, or HES-containing shampoo for 30, 60, and 90 min

Contact time	Shampoo base	Ketoconazole-containing shampoo	HES-containing shampoo
30 min	2.00 ± 0.00	1.00 ± 1.00	1.00 ± 0.00
60 min	2.67 ± 0.58	0.67 ± 0.58	1.00 ± 0.00
90 min	3.00 ± 0.00	1.00 ± 0.00	1.00 ± 0.00

Values are expressed as mean ± standard deviation of CFU counts (×10²) obtained from three independent batches. Statistical comparisons were performed using two-way ANOVA followed by Tukey’s HSD test. A significant overall effect of treatment was detected (p < 0.001), whereas contact time and the treatment × contact time interaction were not significant

Tukey’s HSD test showed that the shampoo base produced significantly higher CFU counts than both the ketoconazole-containing shampoo and the HES-containing shampoo (*p* < 0.001 for both comparisons). No statistically significant difference was detected between the ketoconazole-containing and HES-containing shampoos (*p* = 0.564). Descriptively, the HES- and ketoconazole-containing formulations showed lower mean CFU counts than the shampoo base at all evaluated contact times.

### Preliminary stability evaluation of the HES-containing shampoo

During the 30-day preliminary stability study, no visible changes in color, odor, or homogeneity were observed in the HES-containing shampoo stored at 4, 25, or 40 °C. The pH values remained close to 6.0 throughout the evaluation period under all three storage conditions. The viscosity measured at 25 °C also remained close to 3,000 cP during the study period. Overall, no marked changes were detected in the organoleptic and physicochemical parameters evaluated during the 30-day study.

## Discussion

In the present study, HES inhibited the visible growth of both fungal species evaluated, with MIC values of 125 µg mL⁻¹ against *M. pachydermatis* and 750 µg mL⁻¹ against *T. rubrum*. Under the same experimental conditions, the MIC values of ketoconazole were 112.5 and 900 µg mL⁻¹, respectively. The higher concentrations required to inhibit *T. rubrum* indicate that this microorganism was less susceptible than *M. pachydermatis* to both treatments under the conditions used in this study. However, the numerical proximity between the MIC values of HES and ketoconazole should not be interpreted as evidence of therapeutic equivalence.

According to the interpretative criteria proposed by Fernandez-Andrade et al. [23], natural products with MIC values of up to 0.5 mg mL⁻¹ are classified as potent inhibitors, those with MIC values ranging from 0.6 to 1.5 mg mL⁻¹ as moderate inhibitors, and those with MIC values greater than 1.6 mg mL⁻¹ as weak inhibitors. Based on these criteria, HES would be categorized as a potent inhibitor of *M. pachydermatis* and a moderate inhibitor of *T. rubrum*. These categories are descriptive and should not be interpreted as standardized clinical susceptibility breakpoints. Previous studies have reported considerable variation in the antifungal activity of extracts obtained from *Agave* species. Hammuel et al. [24] reported an MIC greater than 20,000 µg mL⁻¹ for a methanolic extract of *A. sisalana* against *M. pachydermatis*. Verástegui et al. [25] reported MIC values ranging from 2,000 to 6,000 µg mL⁻¹ for extracts from other *Agave* species against *Microsporum gypseum*, *Trichophyton tonsurans*, and *Sporothrix schenckii*. Costa et al. [5] reported inhibition of *Candida albicans* and *M. pachydermatis* by an aqueous extract of *A. sisalana* at concentrations of 87 and 1,400 µg mL⁻¹, respectively. Direct comparisons among these studies should be made cautiously because differences in plant species, plant material, extraction procedures, fungal strains, inoculum concentrations, culture media, and endpoint determination can substantially influence the measured antifungal activity [16, 26]. Nevertheless, the differences among the reported values indicate that extraction and fractionation procedures may affect the antifungal activity of preparations obtained from *Agave* species.

Steroidal saponins and saponin-rich fractions have previously been identified in *A. sisalana* residues [8]. Plant-derived saponins have demonstrated antifungal activity, which has been associated with interactions with fungal membrane sterols and consequent loss of membrane integrity [9, 27]. However, the chemical composition of HES was not determined in the present study. Therefore, the observed antifungal activity cannot be attributed specifically to saponins or to any other chemical class, and the compounds responsible for this effect remain to be identified. Chemical characterization is also required to determine how the hexane-partitioning step influenced the composition of the resulting extract.

The incorporation of HES into a shampoo base allowed its activity against *T. rubrum* to be evaluated in a formulated product. This dermatophyte is an important etiological agent of superficial fungal infections in humans [13, 14]. The shampoo base produced significantly higher CFU counts than both the HES-containing and ketoconazole-containing shampoos, demonstrating an overall treatment effect. No significant effect of contact time or treatment × contact time interaction was detected, indicating that the experiment did not demonstrate a time-dependent change in CFU counts between 30 and 90 min. Although no statistically significant difference was detected between the HES-containing and ketoconazole-containing shampoos, this result indicates only that a difference was not detected under the conditions and sample size employed. It does not establish statistical equivalence between the formulations because the study was not designed as an equivalence or non-inferiority trial. The findings should therefore be interpreted as evidence of in vitro antifungal activity of the HES-containing formulation under the experimental conditions evaluated.

During the 30-day preliminary stability study, no marked changes in color, odor, homogeneity, pH, or viscosity were observed. The pH remained close to 6.0 at all storage temperatures, while viscosity measured at 25 °C remained close to 3,000 cP. These findings indicate that the evaluated organoleptic and physicochemical parameters remained relatively unchanged during the study period. However, the short evaluation period does not establish the long-term stability or shelf life of the formulation. Moreover, maintenance of pH and viscosity alone does not demonstrate cutaneous tolerability or safety for topical application.

The present study has additional limitations. HES was not chemically characterized, and only one reference strain of each fungal species was evaluated. The MIC was determined by the absence of visible growth, without determination of the minimum fungicidal concentration; therefore, the results do not distinguish between fungistatic and fungicidal effects. The shampoo assay was restricted to *T. rubrum*, and no cytotoxicity, skin irritation, permeation, in vivo, or clinical assessments were performed. Consequently, the findings do not establish clinical efficacy or safety. Future studies should include chromatographic and spectrometric characterization of HES, evaluation against a broader panel of reference and clinical fungal strains, determination of fungicidal activity, in vitro safety testing using skin-relevant cell models, validated dermatological safety assays, and extended stability studies before the formulation is considered for clinical or commercial development.

## Conclusions

HES inhibited the visible growth of *M. pachydermatis* and *T. rubrum* under the experimental conditions evaluated, with MIC values of 125 and 750 µg mL⁻¹, respectively. The HES-containing shampoo produced significantly lower *T. rubrum* CFU counts than the shampoo base, while no statistically significant difference was detected between the HES-containing and ketoconazole-containing formulations. This finding should not be interpreted as evidence of equivalence between the formulations. In addition, no marked changes in the evaluated organoleptic and physicochemical parameters were observed during the 30-day preliminary stability study. These findings support further investigation of HES obtained from *A. sisalana* residues as a potential source of antifungal compounds. Chemical characterization, evaluation against additional fungal strains, fungicidal and safety assessments, and extended stability studies are required before the formulation can be considered for clinical or commercial application.

## Data Availability

The datasets generated and/or analysed during the current study are available from the corresponding author on reasonable request.
